# Bacterial Colonies in Solid Media and Foods: A Review on Their Growth and Interactions with the Micro-Environment

**DOI:** 10.3389/fmicb.2015.01284

**Published:** 2015-12-01

**Authors:** Sophie Jeanson, Juliane Floury, Valérie Gagnaire, Sylvie Lortal, Anne Thierry

**Affiliations:** ^1^INRA, UMR1253, Science and Technology of Milk and EggsRennes, France; ^2^AGROCAMPUS OUEST, UMR1253, Science and Technology of Milk and EggsRennes, France

**Keywords:** bacterial colony, spatial distribution, diffusion limitation, porosity, cheese, Growth

## Abstract

Bacteria, either indigenous or added, are immobilized in solid foods where they grow as colonies. Since the 80's, relatively few research groups have explored the implications of bacteria growing as colonies and mostly focused on pathogens in large colonies on agar/gelatine media. It is only recently that high resolution imaging techniques and biophysical characterization techniques increased the understanding of the growth of bacterial colonies, for different sizes of colonies, at the microscopic level and even down to the molecular level. This review covers the studies on bacterial colony growth in agar or gelatine media mimicking the food environment and in model cheese. The following conclusions have been brought to light. Firstly, under unfavorable conditions, mimicking food conditions, the immobilization of bacteria always constrains their growth in comparison with planktonic growth and increases the sensibility of bacteria to environmental stresses. Secondly, the spatial distribution describes both the distance between colonies and the size of the colonies as a function of the initial level of population. By studying the literature, we concluded that there systematically exists a threshold that distinguishes micro-colonies (radius < 100–200 μm) from macro-colonies (radius >200 μm). Micro-colonies growth resembles planktonic growth and no pH microgradients could be observed. Macro-colonies growth is slower than planktonic growth and pH microgradients could be observed in and around them due to diffusion limitations which occur around, but also inside the macro-colonies. Diffusion limitations of milk proteins have been demonstrated in a model cheese around and in the bacterial colonies. In conclusion, the impact of immobilization is predominant for macro-colonies in comparison with micro-colonies. However, the interaction between the colonies and the food matrix itself remains to be further investigated at the microscopic scale.

## Introduction

Bacteria in food products, whether those added as inocula or those naturally present, are always immobilized. They develop as colonies, either on the surface of or embedded within the food matrices and interact with their micro-environment (Hickey et al., 2015). As previously stated (Hills, 2001), the bacterial cells of the colony “consume the nutrients from the surrounding (food) matrix and in return, liberates end-products into the surrounding matrix modifying its micro-environment.”

Bacterial colonies and biofilms are both formed by clusters of bacteria. Whilst published research focusing on biofilms is abundant (Flemming and Wingender, 2010), that based on the bacterial colony is relatively scarce, especially with respect to food. The question remains unanswered whether there are different phenotypes of bacteria making up biofilms and colonies (and especially surface colonies). A biofilm is well-defined as: “a microbiologically derived sessile community characterized by cells that are irreversibly attached to a substratum or interface or to each other, are embedded in a matrix of extracellular polymeric substances that they have produced, and exhibit an altered phenotype with respect to growth rate and gene expression” (Donlan and Costerton, 2002). On the other hand, bacterial colonies are not so well-defined. In this review, a bacterial colony is taken as a clonal group of cells developed either on the surface of or embedded within a gel-type solid (culture medium or food) from which it takes its growth substrates. Unlike biofilms, a colony is limited by size displaying a definite maximum radius ranging between a few μm to a few mm. As the production of extracellular polymeric substances has never been investigated in colonies, we considered not to be a mandatory property. This review will focus only on bacterial colonies and exclude biofilms.

The literature on growth and metabolism of bacteria growing in colonies is scarce. Usually, growth and metabolism of food bacteria (whether desirable or undesirable) are studied in broth media, i.e., in planktonic cultures. However, in order to predict the growth of bacteria in food, it is preferable to perform the study in conditions that closely reflect the natural condition, i.e., in solid model foods. Furthermore, it has been shown that the predictive models of growth built from data taken from liquid cultures are not accurate in describing immobilized growth, especially under stressful conditions that exist in a food medium (Pipe and Grimson, 2008; Skandamis and Jeanson, 2015). Although, the context of most studies cited in this review relates to food, all of them were performed using laboratory media, such as agar or gelatine media, mimicking the growth parameters of food (*a*_w_, pH, NaCl concentration, etc.). It is only very recently that model foods, such as a model cheese, have been used to study the growth of bacterial colonies *in situ*. Both agar/gelatine and foods are matrices in which bacterial colonies can be embedded (submerged colonies), or on which bacterial colonies can attach (surface colonies). However, there is a major difference between agar/gelatine based media and food matrices. Agar/gelatine media are “neutral” matrices because agar and gelatine are not themselves modified by bacteria, whilst food matrices constitute both a structure and a bound substrate for the bacteria. For example, caseins in cheese are a gel-type structure and also provide nitrogen sources to bacteria. This means that food matrices may change by the bacterial activity.

The aim of this review is to describe the growth of colonies by pointing out when and how it differs from the planktonic growth. We particularly discuss the occurrence of variability at the microscopic scale of the physiological states inside the colony, of pH inside and around colonies and of oxygen around the colonies. The diffusion of substrates within the matrix and the access of bacteria to the substrates is also a major concern for the bacterial activity. The second objective is to build concepts on the different situations when growth of bacteria is impacted by the growth in colonies or not, depending on the initial level of population and two other concepts on the different ways of interacting with a food matrix, i.e., “bubble” or “sponge” concepts. Finally, experimental exploration of these two concepts will be examined in model cheese. Furthermore, a large table assembles the main parameters of growth and size of colonies for different experimental culture conditions studied with several bacterial species (Table 1).

**Table 1:** **Summary of the main studies about growth kinetics and distribution (size and neighboring distances) of bacterial colonies, with details of experimental conditions, and main conclusions**.

**Influent parameters**	**Inoculation level or number of cells per colony**	**Experimental/Growth conditions**	***R*_col_ = colony radius** ***R*_bnd_ = boundary radius (μm)** **d_2D_ and d_3D_ = distance 2D or 3D (μm)**	**Maximal growth rate (μ_max_ in h^−1^)** **Generation time (*T*_g_ in h)**	**Conclusions or comments**	**References**
***BACILLUS CEREUS***						
[agar]	10 cfu/ml	BHI 30°C pH 7.2 + D-[6-^3^H] glucose + ^125^I-rhIGF-I (human insulin-like growth factor)	*[agar] = 1%* 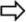 *R*_col_ = **162** *[agar] = 5–7%* 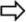 *R*_col_ = **32**		The concentration profiles of both molecules were the same regardless of the [agar]. The colony growth was affected by the reduction of the pore size and the increased strength but not because of a reduced diffusion of nutrients.	Stecchini et al., 1998
***ESCHERICHIA COLI***						
pH Temperature	10^3^−10^4^ cfu/ml	BHI agar Submerged colonies (Gel cassette®)		*Temp. 10°C:* pH 6 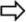 μ_max_ = **0.065** pH 7 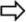 μ_max_ = **0.077** *Temp. 20°C:* pH 5 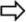 μ_max_ = **0.405** pH 6 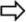 μ_max_ = **0.561** pH 7 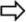 μ_max_ = **0.653** *Temp. 30°C:* pH 5 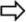 μ_max_ = **1.365** pH 6 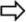 μ_max_ = **1.451** pH 7 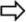 μ_max_ = **1.622** *Temp. 40°C:* pH 5 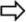 μ_max_ = **1.688** pH 6 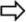 μ_max_ = **1.719** pH 7 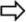 μ_max_ = **1.925**	Only the effect of temperature was significant. Adequate correlation between area and viable counts. Majority of cases (60%) where the model disagree with literature data on foods, the predictions were “fail-safe,” i.e., overestimated.	Skandamis et al., 2007
Size of colonies	1 cfu/ml	Log(surface) = fn(viable cells) 100 pixels = 0.32 mm^2^ = 10^6^ cells/col	*R*_col_ = **160–224** at 11 h *R*_col_ = **746** at 23 h			Mertens et al., 2012
***LACTOBACILLUS CURVATUS***						
Inoculation level	5 cfu/ml 10^2^ cfu/ml 10^3^ cfu/ml	MRS + gelatine 20°C	*R*_col_ = **75** at 150 h *R*_col_ = **190** at 380 h *R*_col_ = **215** at 239 h		Micro-gradients of pH observed for 5 and 100 cfu/ml (intra-colony interactions), but not for 1000 cfu/ml (inter-colony interactions)	Malakar et al., 2000
Inoculation level	*Cells/colony when inter-colony interactions started:*	MRS + agar 30°C	*R*_col_ when inter-colony interactions started:		For pH 7 and pH 6, if *R*_col_ < 200 μm, the equations for growth are relevant; diffusion limitations start when the number of cells in a colony>10^5^	Malakar et al., 2002a
pH	6.10^5^ cells/colony 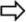 10^2^ cfu/ml		pH 6 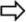 *R*_col_ = **113**			
	10^6^ cells/colony 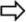 1 cfu/ml		pH 7 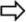 *R*_col_ = **134**			
***LACTOCOCCUS LACTIS***						
[gelatine]	10^3^ cfu/ml	BHIY + glucose 1% ± gelatine 12°C		Broth 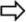 μ_max_ = **0.200** Gelatine: *[gelatine] = 5%* 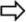 μ_max_ = **0.145** *[gelatine] = 30%* 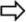 μ_max_ = **0.097**	The increase of [gelatine] decreases the μ_max_ of *L. lactis*, more sensitive than *L. innocua*	Antwi et al., 2007 Theys et al., 2009a
Inoculation level	Inoculation levels (cfu/ml): a- 2.0x10^5^ b- 1.6x10^6^ c- 9.6x10^6^	Model cheese 19°C	a- *R*_col_ = **10.4** (±2.6) b- *R*_col_ = **5.2** (±1.6) c- *R*_col_ = **4.0** (±0.5) a- d_3D_ = **123** b- d_3D_ = **50** c- d_3D_ = **34** Cf. Table 2		Surface between colonies and cheese matrix increased when *R*_col_ decreased: a- S = **1.32** cm^2^/cm^3^ b- S = **5.19** cm^2^/cm^3^ c- S = **9.49** cm^2^/cm^3^	Jeanson et al., 2011
[glucose]	From 10^0^ to 10^6^ cfu/ml	CMR agar 1% 35°C	Cf. Table 2		Colonies up to 10^5^ cells/colony grow with similar growth rate than planktonic cultures, bigger colonies have lower growth rate	Kabanova et al., 2012
[glucose]	10^2^ cfu/ml	M17 ± agar 1% 35°C	*[glucose] = 2 g/l* 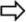 *R*_col_ = **105** *[glucose] = 6 g/l* 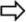 *R*_col_ = **120** *[glucose] = 10 g/l* 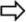 *R*_col_ = **105**		μ_broth_ ≈ μ_agar_ 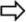 no diffusion limitations for glucose – much less lactic acid produced for the same [glucose] consumed, final pH higher	Kabanova et al., 2013
Planktonic vs. immobilized						
Strain (*n*=6)	A few colonies	Synthetic amino acid medium + glucose 0.5% 30°C	*Surface after 134 h of growth:* From **20** to **165** pixels	*Generation time (min):* From **63** (strain IL1403) to **123**	The proportion of dead cells was related to the surface from 18 to 50%, randomly distributed within the colony	Ryssel et al., 2013
			Rmq: strain IL1403 had the fastest growth Rmq: wide variability of colony size for a same strain	Rmq: same order of strains than for the surfaces		
***NISIN-PRODUCING LACTOCOCCUS LACTIS AND LISTERIA MONOCYTOGENES***						
±CaCO_3_	a- 7.8.10^5^ cfu/ml b- 1.7.10^5^ cfu/ml c- 7.8.10^2^ cfu/ml d- 7.8.10^1^ cfu/ml	BHIY + glucose + agar 30°C	a- d_2D_ = **100** b- d_2D_ = **200** c- d_2D_ = **1100** d- d_2D_ = **5000**		Critical distance (<5000 μm) exists for the inhibition *L. monocytogenes* by the nisin produced by *L. lactis* only if the medium is not buffered	Thomas et al., 1997
***LISTERIA INNOCUA***						
[gelatine]	10^3^ cfu/ml	BHIY + glucose 1% ± gelatine 12°C		Broth 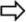 μ_max_ = **0.120** [gelatine] = 5% 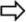 μ_max_ = **0.097** [gelatine] = 30% 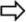 μ_max_ = **0.076**	The increase in [gelatine] decreases the μ_max_ of *Listeria innocua*	Antwi et al., 2007 Theys et al., 2009a
***LISTERIA MONOCYTOGENES***						
[sucrose]	10^3^–10^4^ cfu/ml	TSBY ± gelatine 10°C Submerged colonies (Gel cassette®)		Broth, pH 7 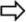 μ_max_ = **0.16** pH 6 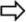 μ_max_ = **0.13** pH 5 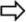 μ_max_ = **0.09** [gelatine], pH 7 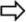 μ_max_ = **0.14** pH 6 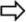 μ_max_ = **0.09** pH 5 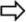 μ_max_ = **0**	Boundaries of growth are narrower 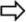 Shrinkage of the growth/no growth regions	Meldrum et al., 2003
pHi						
***SALMONELLA ENTERICA* SUBSP. *ENTERICA* SEROVAR ENTERITIDIS**						
Planktonic vs. immobilized	10^6^ cfu/ml	TSB + glucose 1% + agarose 0.8% pH 7 20°C or 30°C	d_2D_ ≈ **100**	Broth 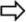 μ_max_ = **0.693** Agar 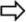 μ_max_ = **0.990**	Faster growth in agar than in broth - physiological heterogeneity within a colony of bacteria growing in a gel matrix	Walker et al., 1998
		*Final population (cfu/ml):* Broth 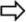 9.3.10^8^ Agar 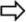 3.4.10^8^ 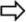 10^2^ cells/colony				
***SALMONELLA ENTERICA* SUBSP. *ENTERICA* SEROVAR TYPHIMURIUM**						
Temperature	10^3^cfu/ml	TSBY + glucose 1% ± gelatine 10%		***Generation times:*** *Temp. 20°C, [NaCl] = 0.5%*, pH 7 Broth 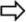 **1.2**; immobilized 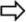 **1.3** *Temp. 12°C, [NaCl] = 0.5%*, pH 7 Broth 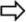 **4.8**; immobilized 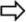 **4.7** *Temp. 20°C, [NaCl] = 0.5%*, pH 4.4 Broth 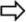 **1.7** immobilized 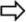 **No growth at 14 d** *Temp. 20°C, [NaCl] = 0.5%*, pH 5 Broth 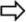 **2** immobilized 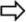 **4.0–4.8** *Temp. 20°C, [NaCl] = 3.5%*, pH 7 Broth 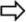 **2**; immobilized 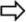 **2.6**	Similar growth in broth medium and immobilized when pH = 7, [NaCl] = 0.5% at 12 and 20°C; The discrepancies between the growth in broth media and the immobilized growth were greater in stressful conditions	Brocklehurst et al., 1995
[NaCl]						
pH						
Planktonic vs. immobilized						
[NaCl]	1 colony/plate	BHI agar 1% 30°C	After 21 h of growth: *[NaCl]* = *0.5%* 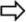 *R*_col_ = **780** *[NaCl]* = *1.5%* 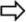 *R*_col_ = **680** *[NaCl] = 2.5%* 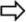 *R*_col_ = **420** *[NaCl] = 3.5%* 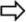 *R*_col_ = **175** *pH = 7* 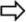 *R*_col_ = **790** *pH = 6* 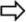 *R*_col_ = **580** *pH = 5* 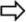 *R*_col_ = **250**	pH 7*, [NaCl] = 0.5%* 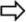 μ_max_ = **0.78** pH 7*, [NaCl] = 2.5%* 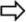 μ_max_ = **0.81** pH 7*, [NaCl] = 3.5%* 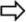 μ_max_ = **0.73** pH 6*, [NaCl] = 0.5%* 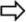 μ_max_ = **0.87** pH 5*, [NaCl] = 0.5%* 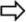 μ_max_ = **0.78**	Decreasing pH and increasing [NaCl] had little effect on growth kinetics except an increase of the Lag phase	McKay and Peters, 1995
pH						
[gelatine]	10^3^ cfu/ml	TSBY + glucose 1% ± gelatine Gelatine 10% for submerged colonies Gelatine 10% or 20% for surface colonies 20°C pH = 7		Broth: [sucrose] = 0% 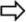 μ_max_ = **0.90** [sucrose] = 20% 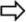 μ_max_ = **0.55** [sucrose] = 30% 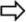 μ_max_ = **0.32** Submerged: [sucrose] = 0% 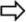 μ_max_ = **0.80** [sucrose] = 20% 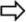 μ_max_ = **0.48** [sucrose] = 30% 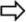 μ_max_ = **0.30** Surface: [sucrose] = 0% 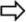 μ_max_ = **0.68** [sucrose] = 20% 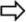 μ_max_ = **0.30** [sucrose] = 30% 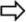 μ_max_ = **0.0**	Whatever the [sucrose], μ_broth_ > μ_submerged_ > μ_surface_ Surface colonies have greater vulnerability to inhibition than submerged colonies, but no effect of [gelatine] up to 20%	Brocklehurst et al., 1997
[sucrose]						
	1 colony/plate	BHI agar 1% pH 7 30°C	*R*_col_ > **200**	Centre of colony 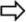 μ_max_ = **0.37** >200 μm from the center 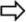 μ_max_ = **0.69**	Regional variations of μ within the colony, μmax outside the colony while μ < μ_max_ in the center of the colony	McKay et al., 1997
Inoculation level	1 cfu/ml	TSB + gelatine 10% (gel cassettes®) 20°C	Inoculation 10^3^ cfu/ml: pH 5 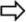 *R*_col_ = **300** pH 7 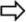 *R*_col_ = **450** Inoculation 1 cfu/ml: pH 5*, [glc] = 0 or 0.1%* 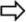 *R*_col_ = **400** pH 5*, [glc] = 1%* 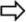 *R*_col_ = **800** *pH 7, [glc] = 0 or 1%* 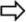 *R*_col_ = **1300** pH 5*, [glc] = 0.1%* 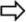 *R*_col_ = **1600**		Acidification is more dependent on the [glucose] than on the inoculation level. Rings of pH (microgradients) were still observed after 5 d when [glucose] was 1% while pH microgradients disappeared when the colony aged 5–6 d when [glucose] = 0.1%	Walker et al., 1997
Initial pH	10^3^ cfu/ml					
[glucose]						
Planktonic vs. immobilized	10^4^ cfu/ml	TSB + glucose 1% + gelatine 10% pH 7 20°C *Final population (cfu/ml):* 2.84.10^9^ 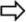 10^5^ cells/colony	d_2D_ = **464**	μ_max_ = **0.550**	Slower growth in gelatine than in broth—physiological heterogeneity within a colony of bacteria growing in a gel matrix	Walker et al., 1998
Planktonic vs. immobilized	10^3^cfu/ml	TBS ± gelatine 20% 20°C	Broth: pH 7 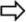 *R*_col_ = **8.8** ±0.1 pH 5 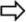 *R*_col_ = **8.6** ±0.2	Broth: pH 7 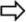 μ_max_ = **0.179** ±0.004 pH 5 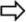 μ_max_ = **0.158** ±0.003 Gelatine: pH 7 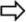 μ_max_ = **0.148** ±0.003 pH 5 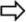 μ_max_ = **0.098** ±0.002	Growth rates are significantly higher in broth but the final counts were similar. Faster consumption of glucose occurred in broth compared in gelatine because of aerobic metabolism	Skandamis et al., 2000
pH		*Final population (cfu/ml):* Broth, pH 7 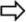 6.3.10^8^ pH 5 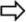 4.10^8^ Gelatine, pH 7 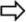 4.10^8^ pH 5 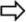 6.3.10^8^	Gelatine: pH 7 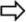 *R*_col_ = **8.6** ±0.3 pH 5 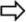 *R*_col_ = **8.8** ±0.2			
	10^3^ cfu/ml	20°C, 10% gelatine, 0.5% NaCl, pH 7 Final population = 10^9^ cfu/ml	*R*_col_ = **90**	μ_max_ = **1.1**	Linear correlation between Log(volume of the colony) and normalized doubling time. The volume continue to increase while Log(cfu/ml) is stable	Wright et al., 2000
Planktonic vs. immobilized	10^3^ cfu/ml	TSB without glucose 20°C Petri dish for gelatine 1% Gel cassettes® for gelatine 5%		Broth: pH *4.5, a_w_ = 0.970* 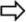 μ_max_ = **0.10** *a_w_ = 0.990* 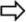 μ_max_ = **0.45** pH *5.5, a_w_ = 0.970* 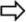 μ_max_ = **0.45** *a_w_ = 0.990* 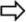 μ_max_ = **0.70** [*gelatine*] = 1%: pH *4.5, a_w_ = 0.970* 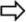 μ_max_ = **0.08** *a_w_ = 0.990* 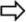 μ_max_ = **0.35** pH *5.5, a_w_ = 0.970* 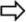 μ_max_ = **0.30** *a_w_ = 0.990* 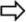 μ_max_ = **0.45** [gelatine] = 1%: pH *4.5, a_w_ = 0.970* 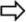 μ_max_ = **0.1** *a_w_ = 0.990* 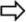 μ_max_ = **0.35** pH *5.5, a_w_ = 0.970* 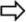 μ_max_ = **0.30** *a_w_ = 0.990* 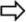 μ_max_ = **0.45**	a_w_ seems to have a higher influence on growth than pH. Planktonic vs. gelatine 1% has an effect on growth but not the increase of [gelatine] from 1 to 5%	Theys et al., 2008
[gelatine]						
pH						
a_w_						
pH	10^3^ cfu/ml	TSB + gelatine 5% Final population = 5.10^8^ cfu/ml	*pH = 5.25, a_w_ = 0.980* 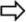 R_max_ = **68** *pH = 4.50, a_w_ = 0.975* 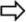 R_30h_ = **54**	pH *5.5, a_w_ = 0.990* 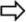 μ_max_ = **0.45** pH *5.25, a_w_ = 0.980* 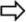 μ_max_ = **0.36** pH *4.50, a_w_ = 0.975* 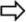 μ_max_ = **0.15–0.19**	Exponential growth until appearance of a dead fraction: 34% at pH 5.25 72% at pH 4.50	Theys et al., 2009b
a_w_						
[gelatine]	10^3^ cfu/ml	a_w_ = 0.099 or [NaCl] = 1.5% pH = 5.5 20°C		*[gelatine] from 0 to 5%, a_w_ = 0.99* 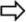 μ_max_ = **0.54** *[gelatine] = 1%, [NaCl] = 1.5%* 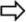 μ_max_ = **0.53** *[gelatine] = 5%, [NaCl] = 1.5%* 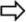 μ_max_ = **0.47** *[gelatine] = 0%, ∀ a_w_ and [NaCl]* 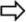 μ_max_ = **0.85**		Theys et al., 2010
Planktonic vs. immobilized	10^3^ cfu/ml	25°C LB ± pluronic acid *Final population* = 10^9^ cfu/ml	*In mid-exponential phase* 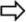 *R*_col_ = **100** *In late stationary phase* 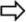 *R*_col_ = **190**	Broth: 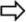 μ_max_ = **0.87** ±0.04 Colonies: 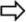 μ_max_ = **0.82** ±0.09	+3 h of lag phase but less virulence in immobilized growth compared to planktonic growth	Knudsen et al., 2012

*Bold values are the measured values in the studies, normal values are the experimental conditions*.

## Historical perspective of the scientific community working on bacterial colonies

As early as the 60's, Pirt, of the University of London, had started to take into account the immobilization of bacteria in the predictive growth models (Pirt, 1967). More recently the 90's, Wimpenny, from the University of Wales, started to study the consequences for bacteria by growing as colonies. Wimpenny et al. (1995), Thomas and Wimpenny (1996b), and McKay et al. (1997) performed studies on pathogenic bacteria, mostly as large colonies (>500 μm), either surface or submerged, on an agar medium. They determined several characteristics of the behavior in colonies comparing with planktonic growth, such as growth rates under different conditions, and pH gradients within and around colonies of different sizes. Before the research on this topic stopped at the University of Wales, Wimpenny collaborated with Brocklehurst (Walker et al., 1997; Wilson et al., 2002) of the Institute of Food Research (Norwich, UK) who was also working on the immobilized growth of pathogenic bacteria. Brocklehurst and his group (Parker et al., 1998; Wright et al., 2000; Meldrum et al., 2003) developed and patented the Gel Cassette System (Brocklehurst et al., 1995). This system has become the ideal tool to study submerged colonies in gelatine and agar media, which was associated with a non-destructive and *in situ* microscopic examination. It comprises a 2 mm thick frame in a PVC sleeve shown to be permeable to gas. The inoculated medium solidifies inside the frame and the immobilized cells develop as colonies within the formed solid gel. Subsequently, Brocklehurst collaborated with Malakar (Wageningen University, Netherlands) who worked on pH microgradients, introducing imaging techniques (Malakar et al., 2000), and on interactions between colonies of lactic acid bacteria (Malakar et al., 2003) and at a later date with Van Impe (Leuven University, Belgium) whose group still works at improving predictive growth models for immobilized pathogenic bacteria in gelatine media (Antwi et al., 2007; Mertens et al., 2012; Boons et al., 2013). Van Impe studied mostly large pathogen bacterial colonies grown in agar or gelatine media and used micro-electrodes to measure pH. More recently, high resolution imaging techniques have allowed the (i) exploration of small colonies (< 100 μm), (ii) measurement of pH down to a resolution of a few μm, and (iii) increasing numbers of monitored parameters like variability of shape, of growth in which single-cell variability, and of metabolism (Bae et al., 2011a; Gonzalez et al., 2012; Knudsen et al., 2012; Koutsoumanis and Lianou, 2013; Ryssel et al., 2013; Vilain et al., 2014). Other research groups have recently compared planktonic and immobilized bacterial growth using molecular techniques to study the difference of gene expression (Knudsen et al., 2012) and protein expression (Knudsen et al., 2012; Vilain et al., 2014). Microcalorimetry has been recently used to study the carbon metabolism at different inoculation levels (Kabanova et al., 2012). The techniques used to study the immobilized bacterial colonies are described in a recent review (Lobete et al., 2015). Imaging fluorescent techniques have allowed the observation of colonies within an opaque matrix such as model cheese. Our group, at the French National Institute for Agricultural Research (INRA, Rennes, France), explores small colonies of lactic acid bacteria (LAB) and their dynamic micro-environment in a model cheese (pH, diffusion of substrates in and around colonies, etc.) in order to better understand the role of LAB during cheesemaking and ripening at the microscopic scale (Jeanson et al., 2011, 2013; Floury et al., 2013, 2015). We also investigated the role of the size of colonies during ripening by combining omics techniques (Le Boucher et al., 2013, 2015a).

## What does immobilization imply for the growth of bacteria?

The growth of colonies has been studied using a qualitative approach and several publications have described how a bacterial colony grew on and within a solid matrix, how they were distributed depending on the inoculation level, and how neighboring colonies interacted with each other either from the same or different species.

### Growth of immobilized colonies

Since the first studies, it has been demonstrated that the growth of bacterial colonies on the surface is a concentric pattern (Wimpenny, 1992). Cell division starts from the initial immobilized cell, with the colony expanding progressively at the periphery thus following a concentric pattern (Wimpenny, 1992; Pipe and Grimson, 2008). In the exponential growth phase, the number of cultivable cells is linearly correlated to the Log(colony volume) (Wright et al., 2000; Theys et al., 2009b) for submerged colonies or to the Log(colony area) (Guillier et al., 2006; Skandamis et al., 2007; Mertens et al., 2012) for surface colonies. Image analysis techniques have thus been proposed to replace the time-consuming plating techniques. The height of a bacterial colony growing on a surface of a medium was modeled as a function of the glucose concentration of the medium. Indeed, the glucose concentration is low on the top of the colony. It has been suggested that the growth of bacteria and the development of pH profiles in and around the colony were determined by the local presence, and diffusion of glucose, in the medium beneath the colony (Wimpenny, 1992). This was the main reason offered to explain why the growth of immobilized cells may be different from that of planktonic cells. It has been demonstrated that most of the mathematical models based on a laboratory broth overestimate the bacterial growth in milk, and even more so its growth in cheese-like media (Theys et al., 2009a).

In conclusion, all the studies on bacterial colony growth have suggested that the growth of colonies (growth rate, final size, and shape) was determined by local concentration of substrates and thus by possible limitations of the diffusion of substrates or end-products in solids (McKay et al., 1997; Walker et al., 1997; Malakar et al., 2002b; Pipe and Grimson, 2008).

### Distribution of colonies: Size of colonies and distances between colonies

**Figure 1:**
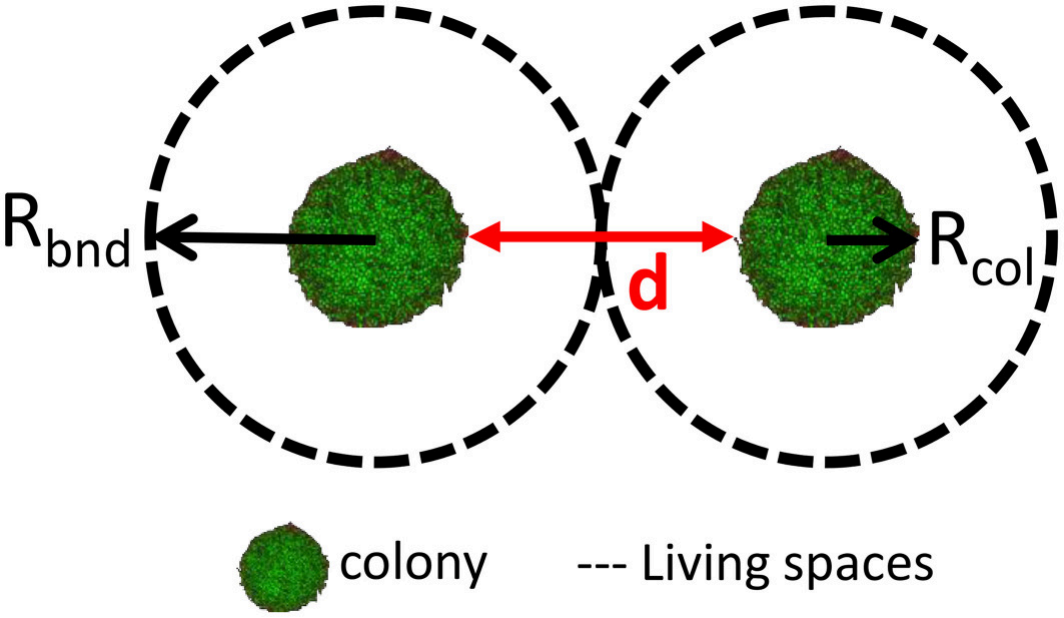
**Representation of the colony and its surrounding “living space” (the area within which the colony is active) with the two respective radii ***R***_col_ and ***R***_bnd_; ***d*** is the distance between two neighboring colonies**. Adapted from Malakar et al. (2002a) and Wimpenny (1992).

When considering the dimensions of a colony, there are two radii of particular importance: the colony radius from the center of the colony to its periphery (*R*_col_), and the boundary radius from the center of the colony to the limit of its influence on the medium (*R*_bnd_) (Malakar et al., 2002a). Figure 1 illustrates these two radii: the colony itself is defined by the radius (*R*_col_) and its “living space” is defined as the region around the colony (*R*_bnd_) within which the activity of the bacterial cells is measurable (dashed line), for example by the consumption of substrates and/or production of end-products. The larger the colony (large *R*_col_), the higher the activity of the colony, the greater the “living spaces” (large *R*_bnd_). Furthermore, the larger the radius *R*_bnd_, the greater the distance for the substrate to diffuse to reach the colony. The value of *R*_bnd_ at the moment of an inoculation of 1 cfu/ml was estimated to be five times longer that for an inoculation of 100 cfu/ml (Malakar et al., 2002a).

The spatial distribution of bacterial colonies is defined by the size of colony and the distances between neighboring colonies. It was measured for the first time in a model cheese, varying with the inoculation levels of a *prt*^−^ strain of *Lactococcus lactis* ranging from 10^5^ to 10^7^ cfu/ml, i.e., within the range used in cheese manufacture (Jeanson et al., 2011). The theoretical distances between colonies were first estimated assuming that (i) all the cells in the inoculum gave rise to a colony and (ii) that they were randomly distributed (Poisson l *a*_w_). These two assumptions were confirmed from experimental data obtained by confocal image analysis. It was also demonstrated that the final population was always the same regardless of the inoculation level (Jeanson et al., 2011). As a consequence, the size of colonies was negatively correlated to the level of inoculation, that is, the lower was the inoculation level, the larger the colonies. The distances between neighboring colonies were 123 and 34 μm for inoculation levels of 2 × 10^5^ and 9.6 × 10^6^ cfu/ml, respectively (Table 1). A separate study (Kabanova et al., 2012), carried out using agar, gave the spatial distribution parameters from experimental data of a larger scale of inoculation levels of a strain of *L. lactis* (from 10^0^ to 10^6^ cfu/ml). The values of distances and radii measured were slightly smaller than those reported by Jeanson et al. (2011) (Table 2). However, the latter used isothermal microcalorimetry which is based on dynamic measurements of heat flow rate. Measurements and calculations of colony radii are mostly in agreement for the strains of the two species (*Lactococcus lactis* and *Streptococcus thermophilus*) reaching final populations over 10^9^ cfu/ml whether they were grown in agar medium or in milk gel/model cheese (Table 2). In the agar medium, the shape of colonies was lenticular; this may explain the difference between the measured and the calculated values. Moreover, the *L. lactis* strain (*lac*^−^/*prt*^−^) producing Green Fluorescent Protein (GFP) used by Jeanson et al. (2011) produced smaller colonies in the model cheese because it reached a lower final population (5 × 10^8^ cfu/ml).

**Table 2:** **Size of colonies (calculated by microcalorimetric method or measured from micrographs) as a function of different inoculation levels of two different species of lactic acid bacteria grown in agar, milk gels, or in model cheese**.

**Inoculation levels (cfu/ml)**	**Agar^a^**			**Model cheese**		**Milk gel^d^**	
	***R*_*col*_ (μm) measured**	***R*_*col*_ (μm) calculated**	**Total number of cells/colony (calculated)**	***R*_col_ (μm) measured^b^**	***R*_col_ (μm) measured^c^**	***R*_col_ (μm) calculated**	**Total number of cells/colony (calculated)**
10^0^	546		1.4 × 10^8^				
10^1^	523 ± 98	150	1.2 × 10^7^			331 ± 1	1.4 × 10^8^
10^2^	192 ± 16	66	2.2 × 10^6^			160 ± 4	1.6 × 10^7^
10^3^	92 ± 18	32	2.5 × 10^5^		55 ± 1	74 ± 0.2	1.6 × 10^6^
10^4^	52 ± 13	14	2.3 × 10^4^		32 ± 4	34 ± 0.5	1.6 × 10^5^
10^5^	25 ± 3	6	1.8 × 10^3^	5 ± 1	16–23	16 ± 0.3	1.6 × 10^4^
10^6^	10 ± 1	3	1.8 × 10^2^	3 ± 1		7 ± 0.01	1.6 × 10^3^
10^7^				2 ± 0.2	4 ± 0.4		

a *Kabanova et al. (2012): Lactococcus lactis subsp. lactis strain, in CRM agar 35°C, R_col_ and total numbers of cells/colony calculated from the microcalorimetric study for a final population between 10^9^ and 10^10^ cfu/ml, R_col_ measured from micrographs*.

b *Jeanson et al. (2011): Lactococcus lactis subsp. cremoris strain producing GFP, in a model cheese, final population measured at 5 × 10^8^ cfu/ml (lac^−^/prt^−^ strain), from confocal microscopy images*.

c *Jeanson et al. (2011), Floury et al. (2015) and Le Boucher et al. (2015b): Lactococcus lactis subsp. idem lactis strain, in a model cheese, final population measured at 5 × 10^9^ cfu/ml (lac^+^/prt^+^ strain), from confocal microscopy images*.

d *Stulova et al. (2015): Streptococcus thermophilus strain, in renneted milk gel, calculated final population of between 1.4 and 1.6 × 10^9^ cfu/ml from a microcalorimetric study*.

*The corresponding total number of cells per colony is also given when calculated in the study*.

For a given inoculation level, the variation of the radii of bacterial colonies followed a Normal distribution centered on the mean radius. Indeed, considering that a colony arises from a single cell, the asynchrony of division of any bacterial culture (Kreft et al., 1998) may explain the variability of the colony radii. Some immobilized cells start their division later than others but all cells stopped to grow at the same time. As a result, different numbers of divisions may occur in neighboring colonies (Koutsoumanis and Lianou, 2013).

### Distances between colonies and interactions between different bacterial species

**Figure 2:**
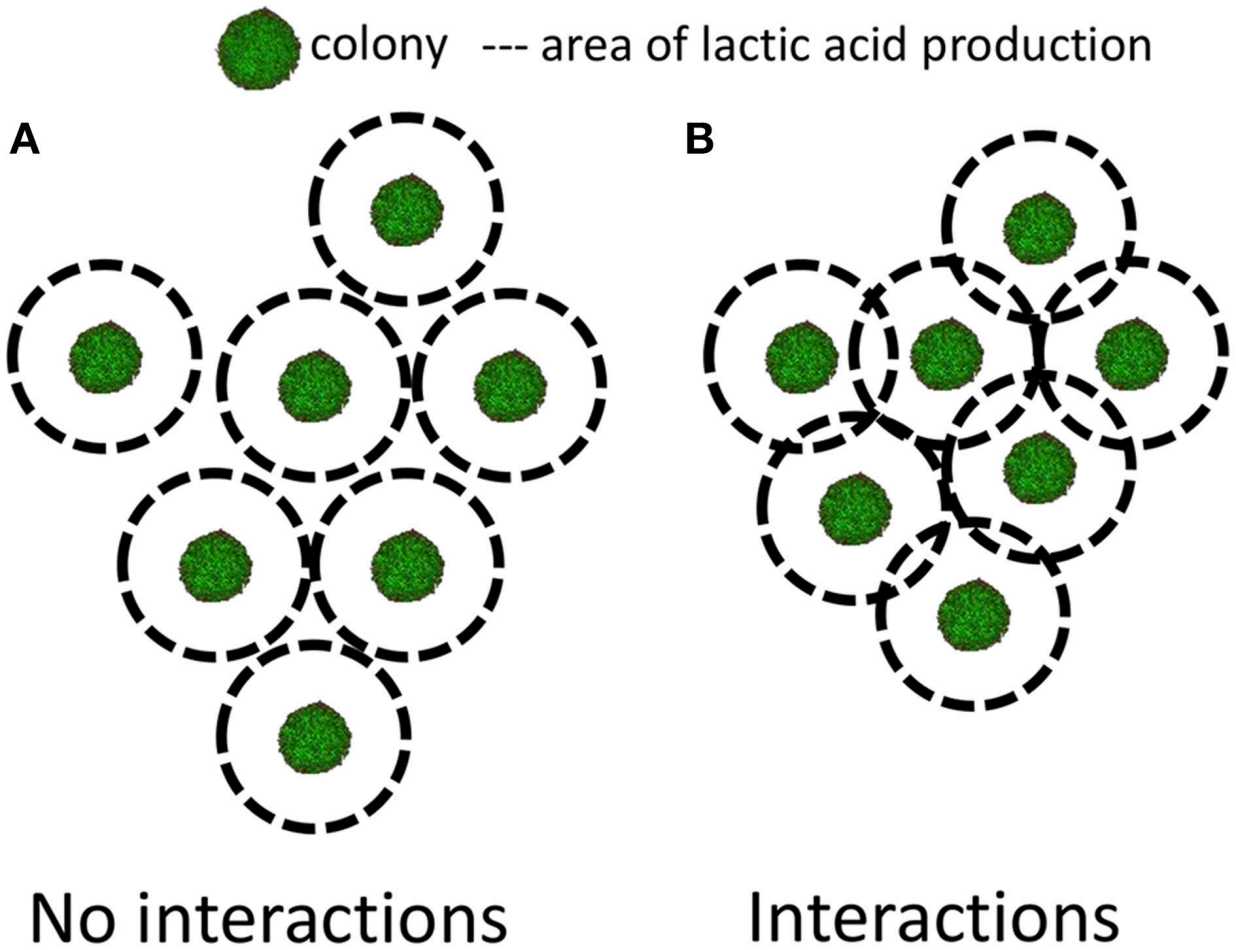
**Representation of two situations of neighboring colonies**. **(A)** When the production of lactic acid of one colony does not impact on its neighbors and **(B)** when the production of lactic acid of one colony does impact on its neighbors. Adapted from Malakar et al. (2002a) and Wimpenny (1992).

If the distance between two neighboring colonies (denoted as *d*) is greater than *R*_bnd_, one can consider that there is no interaction between the colonies, but if it is closer one can consider that some level of interaction exists (Figure 2 and Table 1). This applies whether the neighboring colonies comprise the same strain or are formed from different strains or species. Interactions between different species may be in the form of competition for the same substrate (Thomas and Wimpenny, 1996b) or of inhibition because of production of metabolites such as a bacteriocin like nisin (Thomas and Wimpenny, 1996a) or lactic acid (Antwi et al., 2007). This review focuses on the few studies on colonies taking into account the distances between the inhibiting and the affected colonies. Wimpenny et al. (1995) introduced the concept of “propinquity” defined as the maximum distance between neighboring colonies at which there is still interaction.

A strain of *Salmonella enterica* subsp. *enterica* serotype Enteritidis (named *S*. Enteritidis thereafter) inhibited a strain of *Pseudomonas fluorescens*, while a strain *L. lactis* subsp. *lactis* inhibited a strain of *Listeria monocytogenes* on agar media (Wimpenny et al., 1995). The results showed that the inhibition only occurred if the inoculation level of the inhibiting strain was above 30–100 cfu/ml which corresponded to an average distance between the colonies of 1.4–2.2 mm. These results were then confirmed in another study (Thomas et al., 1997) with strains of *L. monocytogenes* and *L. lactis*. The *Listeria* strain was inhibited either by nisin from a nisin-producer *Lactococcus* strain or, to a lesser extent, by lactic acid production from a non nisin-producer strain. In both cases, the inhibition increased when the distance between colonies of the two species fell from 11 mm to 100 μm. A maximum inhibition distance of 5000 μm was determined, for the inoculation levels of 12 and 4 cells/ml for *L. lactis* and *L. monocytogenes*, respectively, beyond which there was no further inhibition (Thomas et al., 1997).

In conclusion, as low inoculation levels correspond to the formation of colonies far apart (*d* > 1.5–5 mm), it has been suggested that for inoculation levels of 100 cfu/ml and below, no interactions between colonies will occur (Malakar et al., 2000). On the other hand, for an inoculation level greater than 100 cfu/ml, interactions between colonies can be expected (Figure 2).

## Growth in colonies: When and how it differs from planktonic growth

The growth of bacteria as colonies is subjected to several constraints that are absent in planktonic cultures, such as a necessary diffusion of substrates through the solid matrix, with potentially limited access to the substrates. Predictive growth models for bacteria have mainly been based around parameters taken from planktonic cultures and led to the observation that they were not applicable for modeling immobilized growth (Pipe and Grimson, 2008; Skandamis and Jeanson, 2015). Attention was thus given to understand when and how immobilized growth differed from planktonic growth, especially under the stressful conditions of the food environment. In this section, two aspects of the consequences of immobilization of bacteria are presented: (i) their responses to conditions of stress and (ii) on the micro-heterogeneity of the micro-environment inside and around the colonies.

### Narrower boundaries of growth/no growth regions under stressful conditions

The environment existing in food products rarely provides optimal conditions for the growth of microorganisms. The main factors affecting the bacterial growth in food are temperature, pH, NaCl concentration, water activity (*a*_w_) and substrate concentration. Increasing the NaCl or sucrose concentrations also decreases the *a*_w_ and increases the osmotic pressure, with combined negative effects. Several studies have modified these parameters to determine the conditions leading to growth and no growth conditions comparing planktonic and immobilized bacterial growth. Most of these studies have focused on pathogenic species, aiming at predicting or preventing their growth in food. The experimental details and results from the most cited studies in the literature are listed in Table 1.

**Figure 3:**
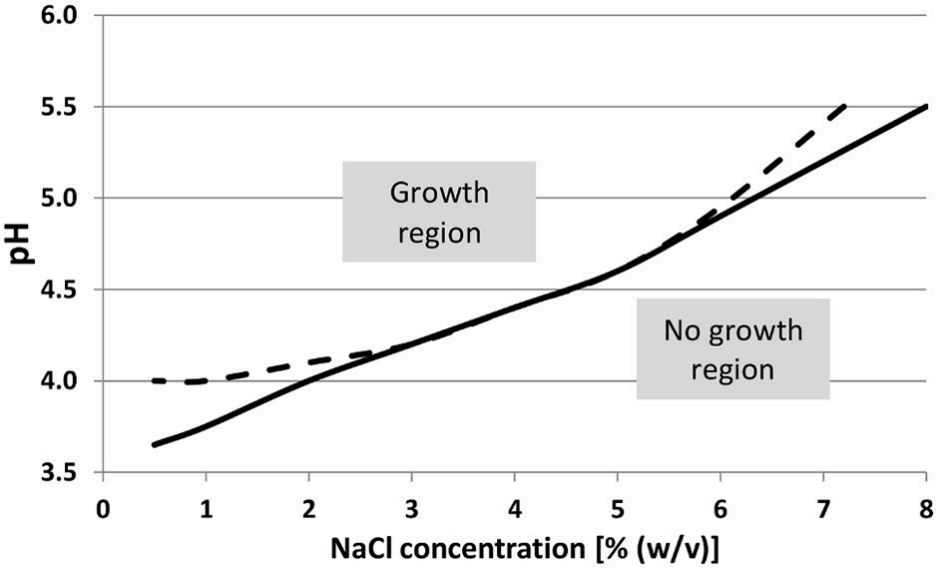
**Growth/no growth regions of ***Salmonella*** Typhimurium in TSB (tryptic soy broth) at 20°C as a function of pH and NaCl concentrations, with gelatine concentrations of 0 and 50 g/l**. Adapted from Theys et al. (2010).

The growth of a strain of *Salmonella enterica* subsp. *enterica* serotype Typhimurium (named *S*. Typhimurium thereafter) in gelatine medium was compared to its growth in broth, at different conditions of pH and NaCl (Brocklehurst et al., 1995). The results show that *S*. Typhimurium behaves the same when growing in colonies and in a planktonic culture when under optimal conditions (pH = 7 and NaCl concentration of 0.5%). However, the generation time *t* (*t* = log2/μ where μ is the growth rate) was increased by a factor between 1.3 and 2 in the more stressful conditions (pH = 5 and NaCl concentration of 3.5%). The growth rates of this bacterial strain were thus ordered as follow: μ_planktonic_ > μ_submerged_ > μ_surface_ regardless of the *a*_w_ when the NaCl concentration was 0.5%, and regardless of the NaCl concentration for maximum *a*_w_ (Brocklehurst et al., 1997). The maximum viable cell counts were less affected by a low value of *a*_w_ reduced by high sucrose and NaCl concentrations if the colony was submerged rather than on the surface. An explanation could be that the substrates are only accessible through the small area of the underside of surface colonies, whilst it is accessible all around the colony on a bigger area when submerged. By comparing a strain of *S*. Typhimurium growing as submerged colonies or in planktonic culture, it was shown that the *a*_w_ was the most influential parameter on the growth rates (Theys et al., 2008). However, decreasing *a*_w_ by increasing NaCl concentration was relatively more harmful to the growth of colonies, because of the combined effect on osmotic pressure, than by increasing gelatine concentrations of the media (Theys et al., 2010). In agreement with the latter, a lower growth rate of growth was observed in planktonic cultures than in submerged colonies of a strain of *S*. Typhimurium and the growth in colonies increased its sensitivity to the inhibition exerted by oregano oil (Skandamis et al., 2000). Surprisingly, the growth rate of submerged colonies of *S*. Typhimurium was found lower in broth than in agar medium, but lower in gelatine medium than in broth (Walker et al., 1998). Furthermore, when the growth rate was not affected by the immobilization of bacteria, the lag phase was increased in comparison to planktonic growth (Knudsen et al., 2012; Nielsen et al., 2013). Figure 3 is an example of the detrimental effect of immobilization of bacteria on their growth when under severe conditions such as low pH and high concentration of NaCl.

**Figure 4:**
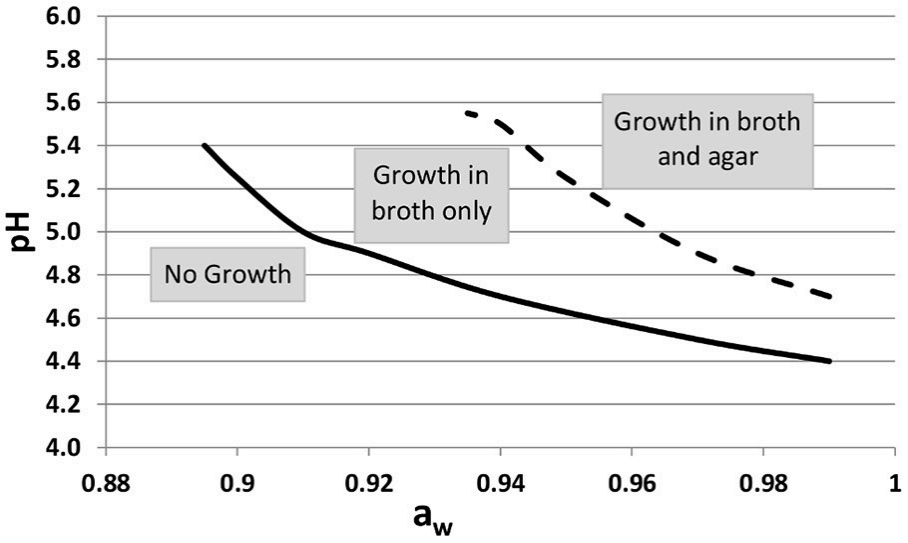
**Growth/No growth regions of ***Listeria monocytogenes*** in broth (solid line) and in agar (dotted line) medium at 25°C as a function of pH and ***a***_w_, (modified by increasing the NaCl concentration)**. Adapted from Koutsoumanis et al. (2004).

Similarly, a strain of *L. monocytogenes* always displayed a lower growth rate when in submerged colonies than in the planktonic form regardless of the sucrose concentration (ranging from 0 to 60%) and the initial pH of the medium. Furthermore, the minimal pH for enabling growth was higher (pH = 5) in colonies than in a planktonic culture (Meldrum et al., 2003). *L. monocytogenes* growth was also shown to be affected by immobilization at low pH and low *a*_w_ (Koutsoumanis et al., 2004) as shown on Figure 4. However, in this study, *a*_w_ had been decreased by increasing the NaCl concentration, the harmful effects of both the NaCl and a low *a*_w_ were thus combined.

The growth of a strain of *Listeria innocua* inoculated at 10^3^ cfu/ml in milk and in gelatinized milk was compared. The growth rates substantially decreased when the concentration of gelatine in the medium was raised from 0 to 50% (Theys et al., 2009a). Under the same conditions, a strain of *L. lactis* was even more detrimentally affected by the increase in gelatine in pasteurized milk (Antwi et al., 2007). The same conclusions were drawn for two LAB strains: the growth rate as colonies was lower than in a broth but only when the inoculation level was lower than 10^3^ cfu/ml for *L. lactis* (Kabanova et al., 2012) or lower than 100 cfu/ml for *Lactobacillus curvatus* (Malakar et al., 2002a). Two studies, using the same *Lactococcus* strain grown in M17 media, showed that, for inoculation levels above 10^2^ or 10^4^ cfu/ml (Kabanova et al., 2012, 2013): (i) there was no glucose diffusion limitation in agar at 1%, (ii) the value of μ_max_ in agar was similar to that in broth during the exponential growth phase, (iii) the LAB strain switched to a heterofermentative metabolism in agar, thus producing less lactic acid with the same amount of glucose, and stopping growth at a higher pH in agar (Kabanova et al., 2013).

The conclusion from all these results is that the growth of bacteria in colonies differs from the planktonic growth, (i) below a specific inoculation level (depending on the species or the strain of bacteria) and (ii) especially in stressful conditions because of narrower boundaries of conditions conducive to growth.

### Heterogeneity in and around colonies (growth, pH, oxygen)

The heterogeneity in and around the colonies results from different aspects of the bacterial activity: growth rates (or lysis), substrate consumption and metabolic activity. The potential existence of microgradients within and around the colony would suggest that the environmental conditions (pH, oxygen, redox potential, etc.) experienced by the cells of the colony are not those of the mean values for the medium (Hills, 2001). The metabolic action, either with respect to the consumption of substrates or the production of end-products, is likely to create microgradients of concentration that cause the heterogeneity of bacterial activity inside the colony. Firstly, the studies about the heterogeneity of growth and metabolic activity inside colonies are discussed. Then, with the technical evolution from micro-electrodes to the recent imaging techniques, the possible existence of microgradients in the environmental parameters inside and around the colony is discussed. These parameters include the pH, resulting from production of lactic acid, and oxygen concentration, resulting from its consumption by bacteria. In order to measure the different types of spatial heterogeneity, all studies were performed on large colonies, mostly on the surface of agar/gelatine media (see Table 1 for details).

#### Heterogeneity of growth rate and metabolic activity between cells of the colony

Two types of heterogeneity within the colony have been shown: (i) a gradient of growth rates or metabolite production from the center to the periphery of the colony arising because of the concentrical growth pattern (Wimpenny, 1992), and (ii) a random heterogeneity due to random differences of division or gene expression between cells (Mikkelsen et al., 2007) which has been observed even in small colonies. Different aspects of the heterogeneity can be observed: morphology, growth rates, or metabolic activity (metabolite pattern).

**Figure 5:**
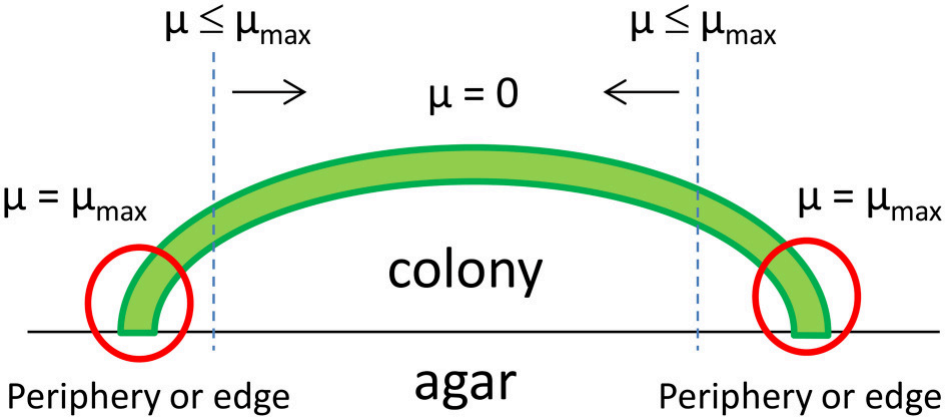
**Simplified model illustrating the spatial variations in the specific growth rate (μ) within a growing bacterial colony of a facultative anaerobe, such as ***Salmonella*** Typhimurium**. Adapted from McKay et al. (1997).

For large colonies, rings exhibiting different morphologies were described (*R*_col_ = 750 μm) for *Escherichia coli* with cells modifying their morphology when aging (Shapiro, 1987), as well as rings with different cell densities for colonies (*R*_col_ = 250–450 μm) of different species of *Bacillus* (Kim et al., 2014). The spatial heterogeneity of colony growth, between active growth for the periphery cells and maintenance activity for the central cells where glucose was scarce, was modeled for *Bacillus* (Kreft et al., 1998). Growth rates were measured in the center and at the periphery of a large (*R*_col_ > 200 μm) colony of *S*. Typhimurium (McKay et al., 1997). Soon after the formation of the colony (13 h), the growth rate at its periphery was twice that of the center, demonstrating that the periphery of a large colony was the region of maximum metabolic activity (Figure 5 and Table 1). The growth slowed down in the center of the colony due to the accumulation of lactic acid possibly combined with the depletion of glucose or carbon sources.

Metabolic heterogeneity has been described by the observation of gradients in lysis activity, as well as gradients of metabolite production or enzyme activity within the colony. An intense lysis of cells was observed in the center of colonies of *Vibrio cholerae* by using a vital stain of the cells (Wimpenny, 1992). Large surface colonies (*R*_col_ ≈ 350 μm) of *Enterobacter cloacae* were sliced (10 μm) from top to bottom, to measure the NADH oxidase activity (Wimpenny, 1992). As oxygen is more available at the surface of the colony, higher activities were found in the upper 100 μm layer. The same conclusion was drawn from using Fourier transform infrared (FT-IR) microspectroscopic mapping of large colonies of *Bacillus megaterium* (obligate aerobes) and *Legionella bozemanii* (microaerophiles). The cells at the top and in the center bottom layers (the “oldest” cells) got the maximum concentrations of capsule components for *B. megaterium* and of poly-β-hydroxybutyric acid, a storage material present in intracellular granules, for *L. bozemanii* (Ngo Thi and Naumann, 2007). In *E. coli* colonies (*R*_col_ = 1 mm), vibrational spectroscopy spectra also showed that for the oldest cells in the surface layers, the RNA level was lower than that in younger cells in the deeper layers (Choo-Smith et al., 2001).

For smaller colonies, results are less clear. For example, the adenylate pool which includes ATP has been shown to be affected by the growth in submerged colonies of *S*. Typhimurium (Walker et al., 1998). The authors suggested that the variation of adenylate production through incubation time, in comparison with broth culture, could be due to an heterogeneity within colonies but this heterogeneity has never been proved. In small colonies (*R*_col_ = 40–60 μm) of *E. coli*, three distinct zones could be observed (center, intermediate and edge) using FT-IR spectra (Ngo-Thi et al., 2003). On the other hand, elastic-scattering patterns of small colonies (*R*_col_ = 50–100 μm) of *L. monocytogenes, E. coli*, and *Salmonella* Montevideo, showed no microgradients of metabolite concentration (Ngo-Thi et al., 2003; Bae et al., 2011a), indicating that their mean profile was representative of the whole colony. Small colonies (*R*_col_ = 25 μm) of *E. coli* have also been shown to be quite homogeneous using vibrational spectroscopy and it was even suggested that 6 h colonies were the most suitable for building an identification data base (Choo-Smith et al., 2001). Identification at early stage of growth of bacterial colonies was possible using a new highly sensitive and non-destructive technique, chromatic confocal microscopy (Drazek et al., 2015).

Finally, the variability of phenotype randomly occurs when a sub-population develops under stressful conditions, either in colonies or in planktonic cultures. This phenomenon was observed under acid stress conditions for small colonies of *L. plantarum* (Ingham et al., 2008) and for *B. cereus* under severe salt stress (den Besten et al., 2007). Heterogeneity of division and shape was observed in small colonies of *L. brevis* (from an initial cluster of a few cells through to several generations) after exposure to an oxidizing disinfectant (Zhao et al., 2014). In small colonies of six different strains of *L. lactis*, the area of dead cells, measured using propidium iodide in microscopy, correlated with growth rates. The dead cells were randomly distributed until 38 h, and were then concentrated in the center of the colony at 134 h (Ryssel et al., 2013).

In conclusion, putting aside the natural random variability of phenotype, these results show, by mapping the growth and the metabolites of large colonies (*R*_col_ > 250 μm), that cells differentiate during the stage of growth within the colony. For this reason, small colonies are homogeneous because all cells exhibit the same growth state.

#### Gradients of pH in and around colonies

The production of lactic acid from bacteria has often been suggested to be the reason why growth stops, due to the accumulation of lactic acid in and around colonies. Using micro-electrodes and then pH-sensitive fluorophores, pH microgradients were recorded only in the case of large colonies, in and around colonies grown on agar/gelatine. However, the question remained if there were also pH microgradients around small colonies or in food such as cheese.

**Figure 6:**
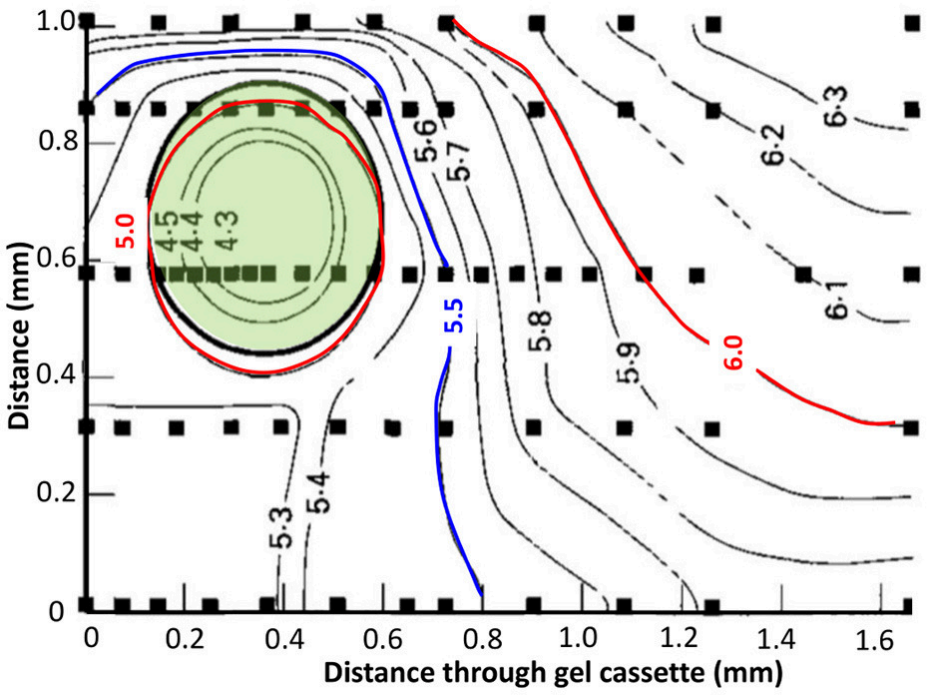
**pH profile through a 2-day old colony of ***Salmonella*** Typhimurium, inoculum density 1 cell/ml, initial pH 7.0, glucose at 1% (w/v)**. Solid squares indicate points where actual measurements were taken. Solid lines indicate pH isopleths which represent an approximation of where the pH gradients may lie. The green area shows colony location. Adapted from Walker et al. (1997).

Using micro-electrodes, the first pH profiles were performed only on large colonies because of the poor resolution of the technique. Microgradients of pH were observed in and around large colonies (*R*_col_ ≈ 10 mm) of *Bacillus cereus* (Wimpenny, 1992) and large surface colonies (*R*_col_ = 800 μm) of *S*. Typhimurium (Walker et al., 1997). Inoculated at 1 cfu/ml with supplementation of glucose at 1%, a difference of 2 pH units was generated between the center of the colony and the edge of the gel (a distance of 1.2 mm); this difference was only 0.5 pH units in a medium supplemented with glucose at 0.1% (Figure 6 and Table 1). For submerged colonies (*R*_col_ = 200 μm) of *S*. Typhimurium in agar gels, Wimpenny et al. (1995) observed a span of pH of 0.8 pH units from the periphery of the colony to the surface of the agar gel (a distance of 3 mm). In contrast, colonies of *S*. Typhimurium inoculated at 10^3^ cfu/ml (*R*_col_ = 200 μm) did not generate measurable pH gradients but modified the pH in the whole bulk medium (Walker et al., 1997). Using ratio-imaging fluorescence, pH microgradients were observed in and around submerged colonies of *L. curvatus* when inoculated at between 5 and 100 cfu/ml (leading to colonies of *R*_col_ = 215 and 190 μm, respectively) but no pH variation was observed when inoculated at 1000 cfu/ml (*R*_col_ = 75 μm) (Malakar et al., 2000).

**Figure 7:**
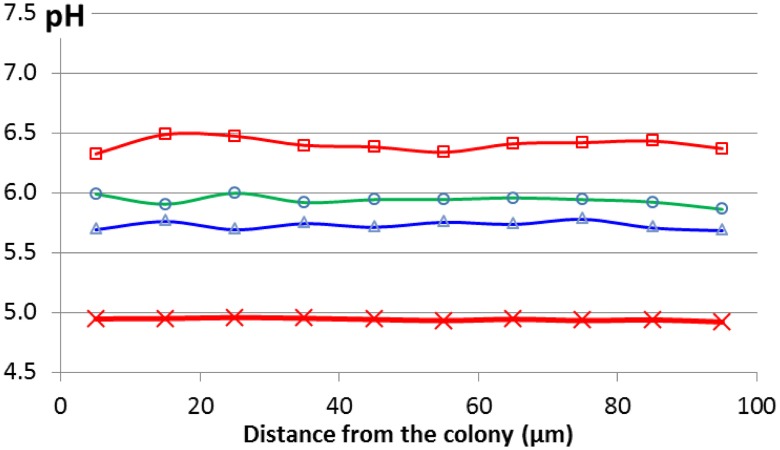
**pH profiles measured using a pH-sensitive fluorophore (C-Snarf-4) and confocal microscopy for a colony (radius = 65 μm) growing in a model cheese throughout acidification: 19 h (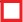), 24 h (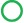), 26 h (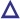), and all measurements from 42 to 72 h (red bold line, 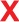)**. Adapted from Jeanson et al. (2013).

In order to confront the observations in agar and gelatine to a real food medium, pH was measured at the microscopic level in a model cheese and in real commercial cheeses. Using ratio-imaging fluorescence, local pH was measured during the acidification of colonies of *L. lactis* whose radii ranged from 17.5 to 55.5 μm, corresponding to the lowest inoculation levels possible in cheesemaking, ranging from 1.3 × 10^3^ to 1.6 × 10^5^ cfu/ml, respectively (Jeanson et al., 2013). Regardless of the observed colony size, no pH microgradients could be observed around colonies (Figure 7). Furthermore, in the same model cheese, the same strain of *L. lactis* displayed no evidence of acid stress at the gene expression level (Cretenet et al., 2011). These results are in agreement with those described above and observed in a gelatine medium for colonies of *L. curvatus* up to 150 μm (Malakar et al., 2000). These consistent results demonstrate that the diffusion of lactic acid was not the limiting factor for growth neither in gelatine nor in a model cheese containing colonies which radius was smaller than 150 μm. Furthermore, in ripened commercial Cheddar cheeses, pH microgradients have been observed at the microscopic scale of a few μm using the fluorescence life-time (FLIM), but not especially around colonies (Burdikova et al., 2015). The accumulation of lactic acid around the colonies has been suggested as the main explanation for the lower growth rate in renneted milk gels when compared with that in liquid milk (Stulova et al., 2015). The simplified composition (no fat, no NaCl) and the homogeneous structure of the model cheese (Jeanson et al., 2013) may explain the non-accumulation of lactic acid around small colonies whilst in commercially available cheeses (Burdikova et al., 2015), lactic acid concentration may vary at the microscopic scale because of a more heterogeneous microstructure.

#### Gradients of oxygen concentration around colonies

**Figure 8:**
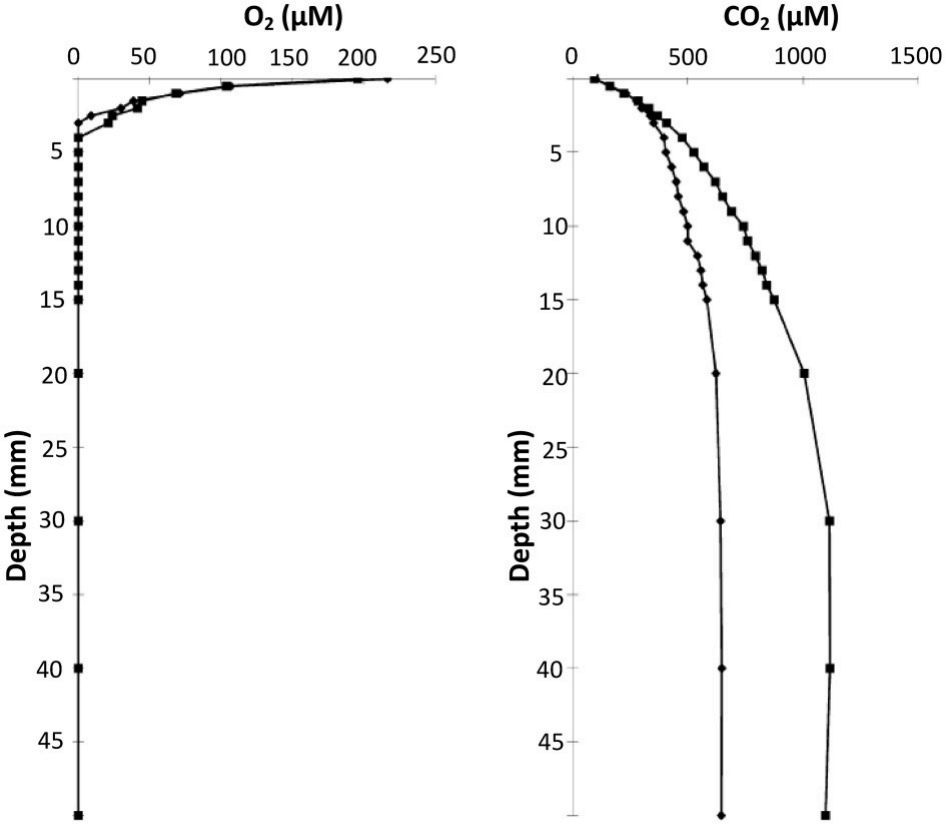
**CO_2_ and O_2_ concentration profiles with depth at 24 h (♦) and 48 h (■) after inoculation with ***Lactobacillus paracasei*** CI3 in MRS 0.1% agar**. A MIMS (membrane inlet mass spectrometric) probe was inserted through column of growth. Adapted from Tammam et al. (2001).

Oxygen (O_2_) is one of the most important parameters for determining the behavior of bacterial growth. Depending on the species, O_2_ can be favorable to growth (aerobes) or inhibiting (anaerobes), or even “neutral” (microaerophilic). For example, for facultative anaerobes such as *S. aureus* or *E. coli*, the cell division has been shown to be more intense on the bottom layer of the colony where O_2_ is scarce and substrates are abundant (Reyrolle and Letellier, 1979). On the other hand, for aerobes such as *Pseudomonas putida*, the top layer of the colony was the zone of the most intense cell division (Reyrolle and Letellier, 1979). Oxygen gradients were first measured inside a colony of *B. cereus* in 1983 using micro-electrodes (Pipe and Grimson, 2008). It has been measured mainly on large surface colonies because O_2_ is present over the whole surface of the colony. The O_2_ concentration decreases with depth moving within the colony and also in depth through the medium below and around the colony in all directions (Wimpenny, 1992). The aerobic zone is considered to exist through 30–40 μm depth in a gelatine medium (Walker et al., 1997). However, Tammam et al. (2001), questions the use of micro-electrodes because they can give non-reproducible results due to the poisoning of the platinum electrodes by other ions. Instead, these authors developed *in situ* mass spectroscopy measurements to investigate the concentrations of O_2_ and CO_2_ concentrations in MRS agar inoculated with a strain of *L. paracasei* (Tammam et al., 2001). Their results show that O_2_ was rapidly consumed by LAB metabolism, while CO_2_ was produced as expected. They showed that in the aerobic zone, there was a gradient of O_2_ concentration through a 5 mm depth in agar after 24 h of inoculation whilst gradients of CO_2_ concentration occurred in the same zone but through a 20 mm depth (Figure 8).

**Figure 9:**
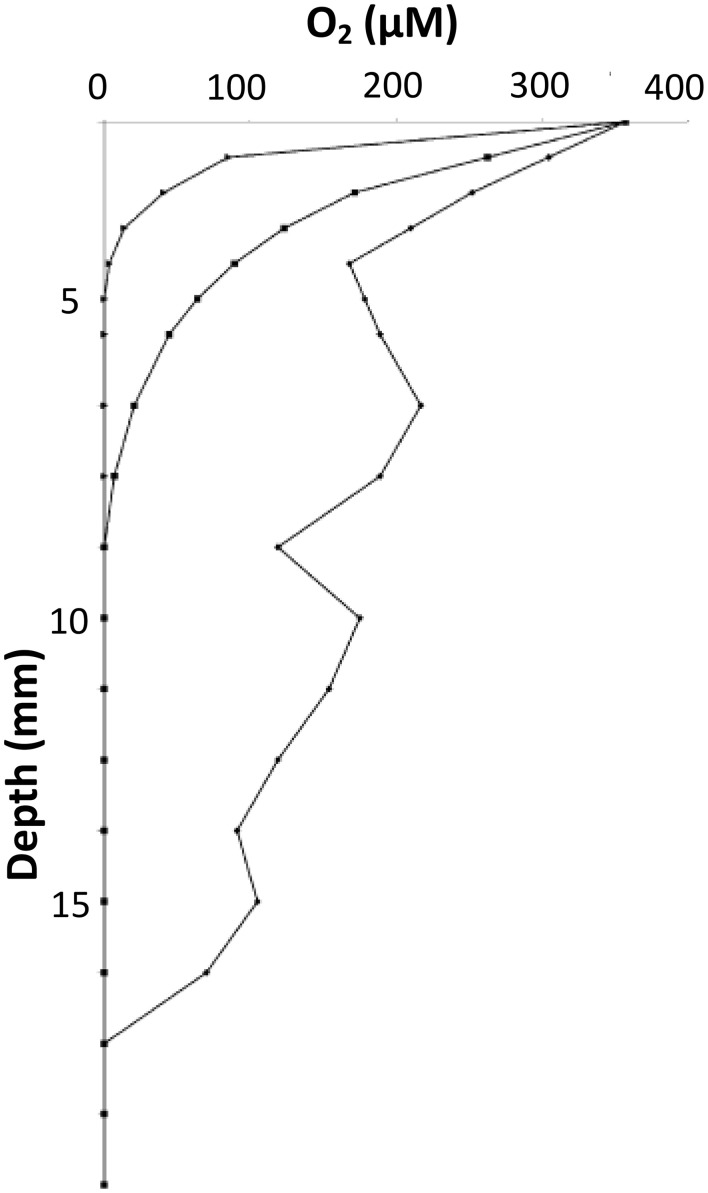
**O_2_ concentration profiles under the rind of Cheddar cheese at 2 days (♦), 9 days (■), and 15 days (▴) of maturation**. Adapted from Tammam et al. (2001).

For the first time in Cheddar cheeses, these authors also investigated the evolution of the concentrations of O_2_ and CO_2_ at depth just below the rind (Tammam et al., 2001). These innovative results concluded that the O_2_ concentration ranged between 350 and 0 μM between the surface of the cheese and 16 mm depth, respectively, after 2 days of ripening. After 15 days, no O_2_ could be measured at a depth of 4 mm (Figure 9). The small colonies of lactococci, observed within the curd by confocal microscopy, were suggested as responsible for the consumption of O_2_ leading to the decrease of the redox potential known in Cheddar cheese manufacture, for example (Caldeo and McSweeney, 2012). The CO_2_ concentration was also directly linked to the heterofermentation of lactococci colonies, which produced up to 16 mM of CO_2_ after 200 days of ripening at a depth of 15 mm. A chemically reducing environment, (i.e., low redox potential), in cheese has been suggested to be essential in the development of flavor and stability (Kristoffersen, 1985). However, in contrast to pH, local variation of the redox potential around colonies has never been investigated at the microscopic scale.

In conclusion, it seems clear that heterogeneity can occur within and around the colonies of bacteria with respect to several parameters directly linked to the bacterial metabolic activity. However, the size of the colonies, and thus the inoculation level, is a major factor determining heterogeneity and the existence of such microgradients.

## Diffusion limitations within the solid matrices

To sustain the growth of bacteria in colonies, substrates have to diffuse from the solid (food) matrix to the colony. At the same time, end-products have to diffuse away from the colony to the matrix, especially if they inhibit bacterial growth such as lactic acid.

The existence of diffusion limitations is the first hypothesis put forth to explain slower growth of the cells in the center of the colony and the microgradients arising in and around the colony. This paradigm has been widely used by different groups to explain their results (Brocklehurst et al., 1997; McKay et al., 1997; Walker et al., 1997; Stecchini et al., 1998; Malakar et al., 2000; Pipe and Grimson, 2008; Kabanova et al., 2012). Even if microgradients of pH and O_2_ have been measured, to our knowledge, microgradients of redox potential, inhibitors, or substrates have not, and their existence is still to be shown. Furthermore, some of these studies initially suggested diffusion limitations of the substrates, but then concluded, in the case of numerous and small colonies in favorable growth conditions, that there were no mass transfer limitations of substrates and lactic acid (Stecchini et al., 1998; Malakar et al., 2002b; Kabanova et al., 2012, 2013). For instance, Malakar et al. (2002b) concluded after they measured the effective diffusion coefficient of lactic acid in gelatine medium that the diffusion of lactic acid was not limiting for growth, and that the growth rate was determined only by the generation time of *L. curvatus*, a LAB strain. They obtained a mean diffusion coefficient of 2.81 × 10^−10^ m^2^/s in MRS with 10% gelatine at 20°C, which they compared with a diffusion coefficient of 1.74 × 10^−10^ m^2^/s in water at 25°C previously measured by Cussler (1997). Therefore, Malakar et al. (2002b) concluded that because both values were of the same order of magnitude in the gel and in the aqueous solution, lactic acid produced by bacteria can easily diffuse through gels. This conclusion is questionable though, because others studies such as those conducted by Ribeiro et al. (2005) and Øyaas et al. (1995) reported values of diffusion coefficient of lactic acid in water much higher than that of Cussler (1997) of between 7 × 10^−10^ and 10 × 10^−10^ m^2^/s at 25°C, meaning that diffusion of lactic acid in the gel media was around 4–6 times lower than in the aqueous solution. It may only be for large colonies (more than 10^5^ cells/colony or 100 cfu/ml), producing a large amount of lactic acid, that mass transfer limitations can be significant (Malakar et al., 2002a). Furthermore, the diffusion coefficient of glucose at 5°C in a 0.8% agar medium was 3.27 × 10^−10^ m^2^/s and was found to decrease linearly with an increase of the agar concentration (Mignot and Junter, 1990). These results demonstrated that the diffusion coefficient of glucose was dependent on the gel microstructure because it decreased with the pore size of the gel network. On the other hand, the diffusion rate of glucose and a small protein (insulin-like growth factor) was shown to be independent of the pore size of the gel with an increased concentration of agar (Stecchini et al., 1998). Finally, the little number of studies on diffusion in gels does not allow clear conclusions on the limiting effect of diffusion of substrates or inhibitors.

In cheese, diffusion of small molecules (water, NaCl, lactose) has been studied while knowledge on diffusion of macro-molecules lacks of data (Floury et al., 2010). Recently, Fluorescence Recovery After Photobleaching was adapted to a model cheese (Floury et al., 2012) in order to measure the diffusion coefficients of fluorescent dextrans of different molecular sizes as well as a range of milk proteins. The major conclusion was that the dextrans (which are flexible and charge-neutral molecules) as large as 2000 kDa were able to diffuse through the model cheese as well as the milk proteins (which are rigid and charged molecules). However, the milk proteins were more hindered in the cheese protein network than dextran molecules of similar hydrodynamic radii (Silva et al., 2013). From these studies, it remains very difficult to draw specific conclusions about the potential effects of diffusion limitations of substrates or end-products on bacterial growth and metabolic activity. Indeed, these diffusion rates have now to be compared to enzymatic reaction rates in immobilized conditions, which are, to our knowledge, still unknown and difficult to determine experimentally. We can only suggest that diffusion within the model cheese matrix is probably not the most limiting factor for the growth of cells at the periphery of colonies where the concentration of the substrates is very high. However, one can wonder what happens to the molecules, especially large molecules, upon reaching the center of the colony. In other words, is the colony porous enough to large molecules, either to penetrate the colony or to be expelled from the colony when released after bacterial lysis?

## Integrated analysis and new concepts of the behavior of bacterial colonies

This section outlines the consequences of the immobilization of bacteria in colonies on their growth and metabolic activity in order to identify general principles and theoretical concepts of importance for fermented food products.

### How the spatial distribution of colonies has a crucial impact on growth

**Figure 10:**
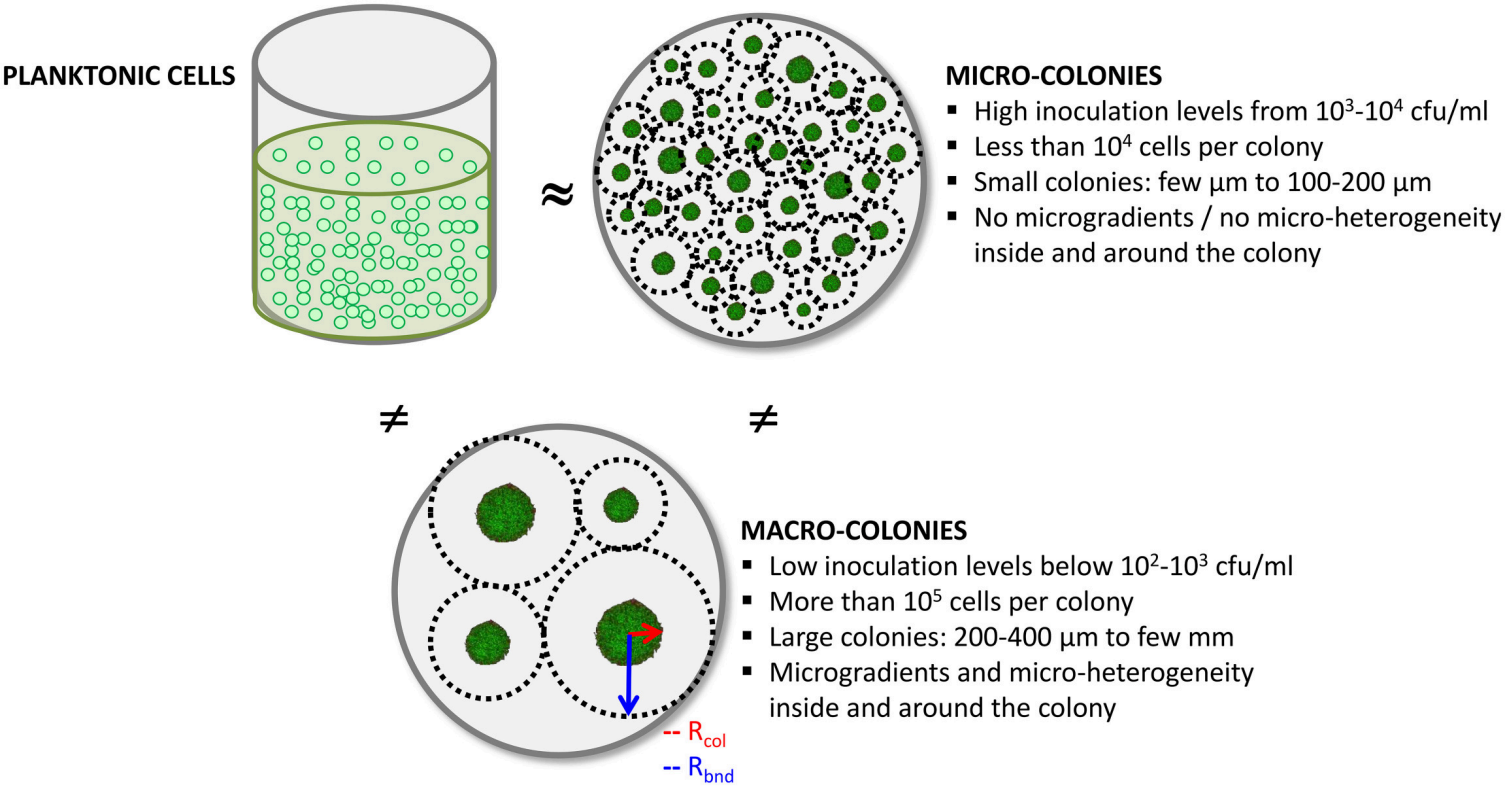
**Schematic diagram of the three culture conditions for bacterial cells and their main characteristics; planktonic culture conditions are the most studied**.

When immobilized as colonies in a solid matrix, bacteria experience multiple constraints on their growth pattern: they develop as colonies and diffusion limitations may limit their access to the substrates. Micro-colonies have previously been defined as colonies displaying a radius *R*_col_ as small as 1.5 μm up to 100 μm (Choo-Smith et al., 2001; Bae et al., 2011b; Zhao et al., 2014) and macro-colonies as those with a radius as large as 2.5 mm (Ngo Thi and Naumann, 2007). However, all these studies were either focused on micro- or on macro-colonies but never integrated data on both. The present overview of literature led to the conclusion that micro- and macro-colonies were two different conditions of growth depending on a threshold of size, determined by the initial level of population. Figure 10 illustrated the two conditions of colonies along with the planktonic form of culture for comparison, defined as follows:

- Large colonies or macro-colonies => colony radii that are generally above a threshold of 100–200 μm (*R*_col_ > 100–200 μm), or typically more than 10^5^ cells per colony, usually generated by inoculation levels or initial populations below 10^2^–10^3^ cfu/ml;
- Small colonies or micro-colonies => colony radii that are generally below 100–200 μm (*R*_col_ < 100–200 μm), or typically less than 10^4^ cells per colony, usually generated by inoculation levels or initial populations above 10^3^–10^4^ cfu/ml.

The threshold between micro-colonies and macro-colonies is determined by the inoculation level above which growth in optimal conditions resembles to planktonic growth. The precise threshold depends on the bacterial species, but implies an inoculation level of between 10^2^ and 10^4^ cfu/ml.

The hypothesis of diffusion limitations around colonies seems relevant for macro-colonies but not for micro-colonies as the growth rate of bacteria is then comparable to that in the exponential phase of planktonic growth (McKay and Peters, 1995; Malakar et al., 2002a; Kabanova et al., 2012, 2013). On the one hand, if micro-colonies on agar/gelatine media display the same growth rate as that for planktonic growth, the most likely hypothesis is that the substrates can penetrate inside micro-colonies so that all the cells have access to the substrates. On the other hand, if there is heterogeneity of growth rates inside the macro-colonies, the hypothesis is that some of the substrates, most likely the larger molecules, do not reach the center of the colony so that those cells cannot access such substrates. These hypotheses lead to the question: are colonies porous to large molecules?

### Two possible concepts for the interactions between a colony and the surrounding matrix: “bubble” or “sponge”

**Figure 11:**
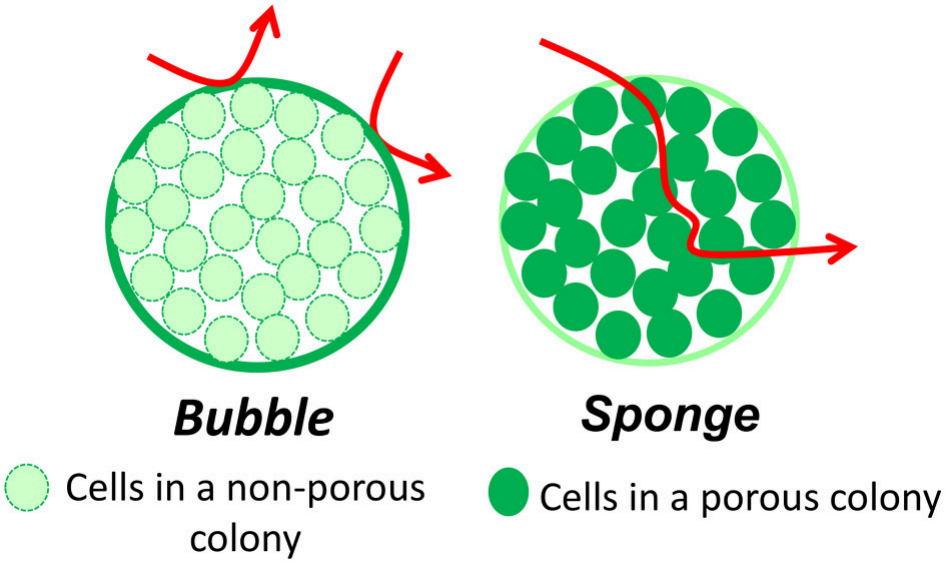
**Schematic representations of the two concepts of interactions between the colony and the matrix; arrows show the diffusing molecules**.

We imagined two extreme concepts of the colony (Figure 11): (i) the colony acts as a “bubble” impermeable to molecules and only the periphery cells are in contact with all of the substrates available from the matrix, (ii) or the colony acts as a “sponge” permeable to all the molecules, representing both substrates and end-products which migrate freely through the colony.

If we consider first the “sponge” scenario, the colony is then a group of individual cells all in contact with its micro-environment. The exchange between the micro-environment and the colony is thus that of each of the cells and depends neither on the size of the colony, nor on their number. This concept is close to the planktonic condition in term of interaction of bacteria with the medium. On the contrary, in the “bubble” scenario, the colony can be considered as a tight cluster of cells and only those at the periphery of the colony are in contact with the micro-environment. Thus, for a given number of bacteria, the total exchange area is then determined by the size and the number of colonies, and is of major importance in governing the activity of the colonies within the matrix. The exchange surface (overall exchange surface per unit of medium volume) increases with the number of colonies as their size decreases (Jeanson et al., 2011). The activity of the colonies within the matrix will thus be increased by an increasing exchange surface if the colonies behave as in the “bubble” scenario whereas there will be no effect if colonies behave as in the “sponge” one. As a consequence, in the “bubble” concept, two different inoculation levels will result in two different values for the exchange surface, and thus two different activities for colonies of different sizes containing the same total number of cells. In the case of two different spatial distributions, labeled 1 and 2, the terms S_1_ and S_2_ represent two different exchange surfaces resulting from the two different inoculation levels I_1_ and I_2_. We assume that (i) the packing density of cells and the volume of individual cells inside the colonies are equal for both spatial distributions; (ii) the inoculation level is equal to the number of colonies (one cell gives one colony). Theoretically, for the same final populations, the ratio S_1_/S_2_ follows the following equation:

$${\text{S}}_{1}/{\text{S}}_{2}={\left({\text{I}}_{1}/{\text{I}}_{2}\right)}^{1/3}$$

**Figure 12:**
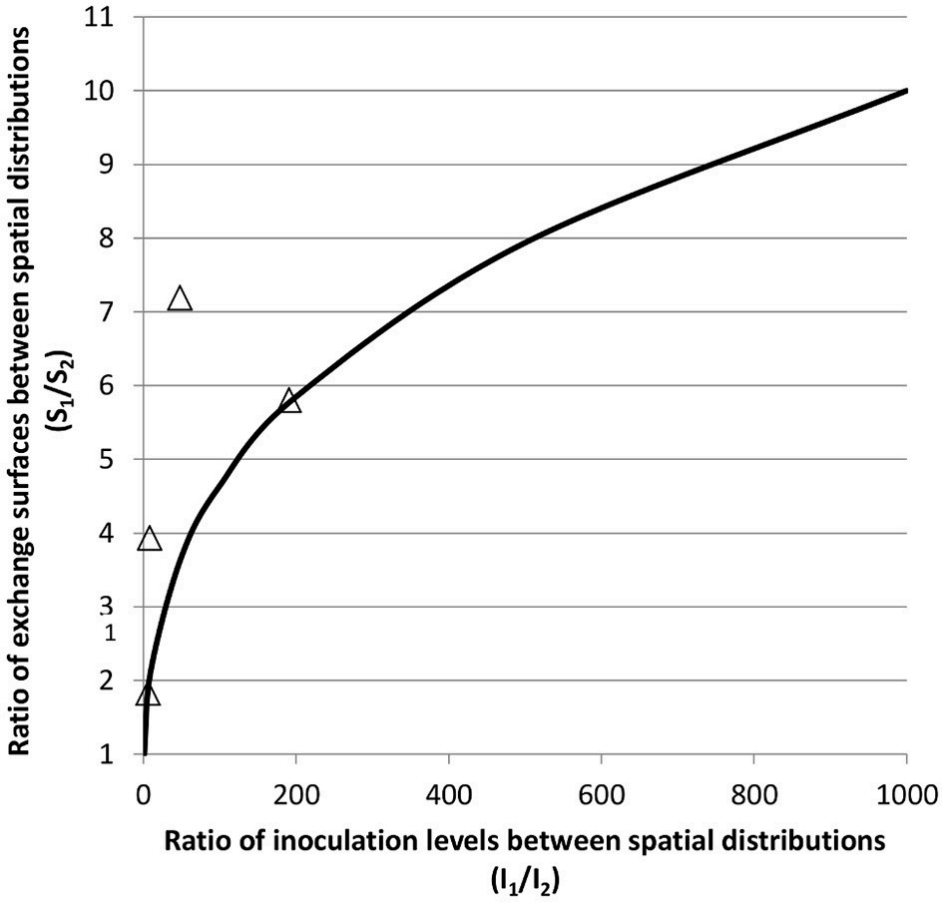
**Theoretical relation (black line) for two different spatial distributions, 1 and 2, between the ratio of the exchange surfaces (S_1_/S_2_) and the ratio of inoculation levels (I_1_/I_2_); (Δ) experimental data either manually measured or obtained from image analysis of confocal microscopy images from Jeanson et al. (2011) and from Le Boucher et al. (2013)**.

However, as seen on Figure 12, the experimental data tend to overestimate the ratio of exchange surfaces (S_1_/S_2_) for a given ratio of inoculation levels (I_1_/I_2_) when compared to the theoretical model. The low precision of the experimental measurements may explain this difference. These concepts are theoretical but may be of great value in food processing. It is thus very important to experimentally explore the question: is the colony functioning as a “bubble” or a “sponge”?

### Experimental exploration of the two concepts “bubble” and “sponge”

#### The porosity of colonies with respect to different types of molecules

As described above, milk proteins and dextrans molecules up to 2000 kDa can diffuse within in a model cheese, but are these large molecules able to also diffuse in to the colony?

A first study explored the resistance to diffusion exerted by cells of *E. coli* and *Rhodospirillum rubrum* homogeneously immobilized in agar through which solutions of glucose and L-malic acid could diffuse (Mignot and Junter, 1990). The results showed that the diffusion of glucose and L-malic acid was negatively and linearly correlated with the increase in cell density in the agar, with an increasing resistance to diffusion over the range from 10^4^ to 10^8^ cells/ml, probably due to the increased tortuosity imposed by the higher density of cells. Considering the packing density of cells in a colony of about 10^11^ cells/ml (Malakar et al., 2002a), the resistance to diffusion of molecules within the colony might be expected to be even higher than for a cell density of 10^8^ cells/ml. We thus investigated the porosity of a bacterial colony to molecules of different sizes (Floury et al., 2013). The results showed that dextran molecules from 4 to 155 kDa could penetrate through lactococcal colonies (*R*_col_ = 15−55 μm) immobilized in an agar gel and in a model cheese. Indeed, dextran molecules as big as 155 kDa (larger than milk proteins) can diffuse through a bacterial colony but their diffusion coefficient could be limited by their size. On the other hand, milk proteins such as bovine serum albumin, lactoferrin and α_s1_-casein did not penetrate inside the lactococcal colonies in a model cheese (Floury et al., 2015). We observed the same results with strains of *Lactobacillus rhamnosus* and *L. plantarum* (Jeanson, personal communication).

In conclusion, it was clearly demonstrated that the diffusion behavior of macromolecules through bacterial colonies immobilized in a model cheese did not depend so much on the size of the diffusing solute molecules, but mainly on their physicochemical properties (Floury et al., 2015). The colony acts like a “sponge” for the neutral and flexible dextran molecules whilst the colony acts like a “bubble” for all the tested proteins.

#### Consequences of the porosity of bacterial colonies in food fermentation: Example of cheese

In cheese, carbon sources such as lactose are soluble and can diffuse freely as in agar or gelatine medium. On the contrary, nitrogen-based substrates are mostly caseins which are bound up in the network and cannot diffuse, except for a minor proportion of free caseins. Assimilable nitrogen substrates are peptides produced from the activity of bacterial cell-wall proteases. In the case of colonies embedded within cheese, only the cells on the periphery can theoretically access the caseins in the network. Taking cheese as an example, this raises the questions: (i) how does the spatial distribution of colonies influence the bacterial metabolism and (ii) how do the cells at the center of the colony access the nitrogen substrates, i.e., the caseins and the casein-derived peptides. If caseins are bound up, one might expect the “bubble” scenario but could the colony act as a “sponge” with respect to the peptides? In order to explore this hypothesis, we measured the influence of two different spatial distributions of micro-colonies of *L. lactis* on the cheese metabolomes during ripening. The inoculations levels, respectively, 1.6 × 10^5^ and 3.1 × 10^7^ cfu/ml thus I_1_/I_2_= 191, generated two sets of model cheeses called *small colonies* cheeses with *R*_col_ = 3.9 ± 0.2 μm and *big colonies* cheeses with *R*_col_ = 26.8 ± 0.2 μm (Le Boucher et al., 2015a). For exactly the same lactococci viable population in the two sets of cheeses, the results showed that lactococci distributed as “small” colonies tended to accelerate proteolysis during ripening in comparison with “big” colonies. As a consequence, *small colonies* cheeses contained higher amounts of amino acids and some of the peptides than *big colonies* cheeses. Nevertheless, the increase in concentration of metabolites between *small* and *big colonies* cheeses ranged from 1.2 to 2.0 for a ratio of S_1_/S_2_ equal to 5 (Le Boucher et al., 2015b). Under the hypothesis of a “bubble” scenario, there should have been an increase in the metabolite concentration close to the ratio S_1_/S_2_, i.e., 5. Under the hypothesis of a “sponge” scenario, there should not have been observed any changes between the proteolysis of the two different spatial distributions. The results obtained with the *small* and *big colony* cheeses are in agreement with the observations on the porosity of colonies. They suggest that the colony acts either as a “sponge” or a “bubble” according to the diffusing molecules, and that some molecules such as peptides may diffuse inside the colony reaching the cells located in the center of the colony. The relatively small proportion of cells in the periphery could produce enough peptides for the cells of the whole colony. It has been previously demonstrated for milk that 10% of a *prt*^+^ strain of *L. lactis* could sustain the growth of 90% of the isogenic *prt*^−^ strain (Juillard and Richard, 1994). Another study performed in a renneted milk gel showed that the overall concentration of total free amino acids was 1.15 times higher than in liquid milk, both being inoculated with a strain of *Streptococcus thermophilus* at 10^5^ cfu/ml (Stulova et al., 2015). Even if rennet increased the hydrolysis of caseins into peptides, this result supports the idea that peptides diffuse inside the colony where they are further degraded into amino acids by intracellular aminopeptidases. Furthermore, for inoculation levels from 10^2^ to 10^6^ cfu/ml, the growth rates were similar in renneted milk gel and liquid milk during the first exponential phase when the bacteria use the non-protein nitrogen sources initially present in the milk. However, the growth rates were subsequently lower in the milk gels than in milk during the second exponential phase when the bacterial strain had to synthesize its own cell-wall protease to sustain growth (Stulova et al., 2015). The hypothesis given by the authors was that there was an accumulation of lactic acid around colonies. However, the fact that the total number of bacteria was 13% higher in the milk gels than in milk at the end of the exponential growth phase, demonstrates that the growth rate was slower but growth lasted longer (Stulova et al., 2015). This result also supports the argument of a limited access to caseins, (leading to the slower growth rate), but to a free access to the peptides.

In conclusion, the interaction of the colony with its surrounding matrix is extremely complex and there are no simple mechanisms that describe how and when the “sponge” and “bubble” conceptions apply. Most likely, the colony acts as a selective filter depending on the properties of the diffusing molecules with a greater preference for flexible and neutral molecules regardless of their size.

## Conclusions

The objective of this review was a comprehensive understanding based on published literature of the impact of bacterial growth as colonies in a food context. Overall, the term “bacterial colonies” embrace different situations depending on the spatial distribution of colonies (size and number of colonies) in the matrix. Finally, the spatial distribution emerges as the most crucial parameter in determining whether the immobilization of bacteria has an impact or not. The conclusions differ widely: (i) if colonies are small and numerous (micro-colonies), the implications of growing in colonies rather than as free planktonic growth are minor; (ii) whereas if colonies are large and relatively few in number (macro-colonies), the implications of such immobilization become significant, mostly in terms of a relatively lower growth rates and their lower resistance when under conditions of stress. In the case of bacterial contamination or indigenous microflora, the initial population is low and colonies thus develop as macro-colonies. It is thus important to increase the understanding on the behavior of pathogenic bacteria in solid matrices in order to improve the predictive growth models in solid foods. In the case of LAB in fermented foods, the inoculation levels are high and one can wonder if the growth in micro-colonies really impacts on the growth and the metabolic activity of bacteria in foods by comparison with that as planktonic growth. However, in fermented foods, the interactions between bacterial colonies and the food matrix itself remain unexplained and inadequately studied using agar/gelatine media. Moreover, interactions and even communication between colonies, like quorum sensing, is still unexplored in solid food media (Skandamis and Nychas, 2012). The newly available imaging techniques may open a great field of research in this respect.

## Author contributions

SJ: design and wrote the review manuscript. JF: expert in the diffusion of molecules in cheese and porosity of colonies; improved the review manuscript. VG: expert in proteolysis by bacteria; improved the review manuscript. SL: initiated the topic in the lab; improved the review manuscript. AT: head of the research group; design and extensively improved the review manuscript.

### Conflict of interest statement

The authors declare that the research was conducted in the absence of any commercial or financial relationships that could be construed as a potential conflict of interest.
